# Analysis of the Use of Carrots, Cauliflower and Broccoli Waste Materials as a Matrix for Thiamine

**DOI:** 10.3390/foods15050801

**Published:** 2026-02-24

**Authors:** Krystyna Eleonora Szymandera-Buszka, Agata Jankowska, Paweł Juszczak

**Affiliations:** 1 Department of Gastronomy Science and Functional Foods, Faculty of Food Science and Nutrition, Poznań University of Life Sciences, Wojska Polskiego 31, 60-624 Poznań, Poland; agata.jankowska@up.poznan.pl; 2 Department of Human Nutrition and Dietetics, Faculty of Food Science and Nutrition, Poznań University of Life Sciences, Wojska Polskiego 31, 60-624 Poznań, Poland; pawel.juszczak@up.poznan.pl

**Keywords:** thiamine, thiamine carriers, fortification, vegetable, zero waste

## Abstract

The investigation aimed to use selected waste plant materials as thiamine matrices for fortification purposes. Thiamine hydrochloride (TCh) and thiamine pyrophosphate (TP) constituted the sources of thiamine. The waste vegetable variables (carrots (crowns, peel), cauliflower, and broccoli (stems, leaves)) were used as a matrix for the thiamine. The peeled carrots, without crowns, as well as the florets of cauliflower and broccoli, were also used as a matrix for thiamine, serving as a reference for the waste used. Fortification effectiveness was analysed based on thiamine content analysis in the product immediately after freeze-drying and after storage (230 days at 4, 21, and 40 °C). The results confirmed that after six months of storage, these products contained thiamine at 55 to 90% of the level found in samples immediately after drying. The results confirm the effectiveness of using analysed waste plant materials as matrices for thiamine. The highest effectiveness was confirmed for broccoli and cauliflower leaves. The analysis of the influence of all predictors on thiamine changes revealed that storage temperature significantly affected thiamine loss in all carriers. It was confirmed that the lower the storage temperature, the lower the dynamics of thiamine loss. It was also confirmed that TP had a lower thiamine retention.

## 1. Introduction

As consistently recommended by the World Health Organization (WHO), consuming adequate levels of fruit and vegetables is one of the fundamental elements of a healthy diet [1]. Vegetables are a rich source of nutrients and bioactive compounds, including vitamins, minerals, and phenolic compounds [2,3]. Numerous studies have demonstrated that sufficient vegetable intake is associated with substantial health benefits, such as improved gastrointestinal and visual function and a reduced risk of certain cancers, cardiovascular diseases, stroke, and other chronic conditions [4]. Despite these benefits, vegetable consumption remains below the recommended levels in many populations. The World Health Organization recommends that adults consume at least two portions of fruit and three portions of vegetables per day [5]. However, vegetable consumption remains too low. Adults typically need 2 to 3.5 cups of vegetables per day, but the exact amount depends on age, sex, and activity level. Achieving these goals involves eating a variety of colours and types of vegetables and incorporating them into every meal and snack [6].

Food fortification and supplementation are the most commonly used strategies to alleviate micronutrient and vitamin deficiencies among humans [7,8,9]. Fortifying foods with vitamins can lead to higher intakes from food products. This can ultimately reduce the need for dietary supplements. Studies have demonstrated the effectiveness of vegetables as a nutrient matrix. The inclusion of plant-based foods in the diet is also associated with increased fibre intake and related health benefits [9]. Research indicates that adequate dietary fibre intake positively influences the composition and activity of the gut microbiota, enhances intestinal motility, insulin sensitivity, and overall metabolic health, while reducing the risk of colorectal cancer and cardiovascular disease [10,11,12]. Vegetables such as pumpkin, broccoli, carrots, and cauliflower are an important natural source of dietary fibre [12,13,14]. Fortification of vegetables may also constitute an attractive alternative source of thiamine for consumer groups. Thiamine is a rate-limiting cofactor for many enzymes, including those involved in glucose, fatty acid, and amino acid pathways. Thiamine deficiency is still being observed in both poor and highly developed countries [15,16].

Thiamine deficiency is associated with disease-related malnutrition, gastrointestinal disorders, chronic use of diuretics (for treating heart failure), adherence to monotonous or restrictive diets, and increased metabolic demand [13,16]. Vitamin B1 deficits may be common in children, the elderly, and physically active young people, as well as pregnant women. In neonates and infants, thiamine deficiency can result in severe neurological and cardiovascular complications, including peripheral neuropathy, heart failure, and impaired neurodevelopment, and is associated with a high risk of mortality if left untreated [17,18]. Owing to the vitamin’s short half-life, deficiency may manifest rapidly in exclusively breastfed infants, leading to acute and potentially life-threatening clinical outcomes [17]. In older adults, inadequate thiamine status has been associated with impaired cognitive function and an increased risk of neurodegenerative disorders, including Alzheimer’s disease, reflecting its essential role in energy metabolism and neuronal integrity. Consistently, patients with thiamine deficiency have been reported to exhibit a higher prevalence of Alzheimer’s disease, depression, and heart failure, as well as an increased risk of falls and stroke, indicating the potential importance of adequate dietary vitamin B1 intake in ageing populations [19,20,21]. Moreover, thiamine deficiency may promote peripheral neuropathy and neuroinflammatory processes, thereby exacerbating functional decline and neurological symptoms in elderly individuals [22,23,24]. Thiamine deficiency is a well-established cause of peripheral polyneuropathy, leading to axonal degeneration, sensory disturbances, and motor dysfunction due to impaired neuronal energy metabolism, and has been reported across all age groups depending on dietary intake, metabolic demands, and underlying health conditions [13,21,23,24,25,26].

Thiamine must be regularly supplied to the human body in adequate amounts with food [27]. Prevention of thiamine deficiency involves a multi-pronged approach, including patient education on diet and lifestyle, as well as dietary modifications incorporating thiamine-rich foods or fortified products [28,29,30]. The search for new matrices for thiamine aims to increase the efficiency of its application to food. Adding the active ingredient at low concentrations (e.g., in milligrams) first to the matrix and then adding at the higher concentration (e.g., 30%) to the food allows for its even mixing with the food. This ensures that each serving of the food product contains the intended amount of thiamine. Preliminary studies have confirmed the possibility of using pumpkin, broccoli, and cauliflower as the matrix for thiamine [31]. In the referenced study, these vegetables were fortified with thiamine hydrochloride and thiamine pyrophosphate and were found to retain approximately 75–89% of the added vitamin. The results demonstrated that the vegetables maintained a substantial proportion of thiamine during processing and storage, confirming their potential as vehicles for food fortification [31]. These results also supported the possibility of adding these preparations to flour products and enriching them with thiamine. However, the study used only edible vegetable parts, such as pumpkin flesh and cauliflower and broccoli florets, whereas other anatomical components were discarded. These vegetables are among the most widely consumed globally and are extensively processed in both household and industrial settings [32,33,34,35]. As a result, their processing generates substantial amounts of by-products, including stems, leaves, crowns, and peels, which often account for approximately 30–50% of the fresh biomass [36,37,38,39]. These discarded parts represent a significant underutilised resource with potential applications in food fortification, functional foods, or as matrices for bioactive compounds, contributing to Zero Waste strategies and sustainable food systems [40,41]. Utilising such vegetable residues can improve nutrient recovery, reduce environmental impacts, and generate economic benefits by valorising materials that would otherwise be discarded [3,32,39,42,43].

Innovative food technology is increasingly focusing on the utilisation of plant by-products and waste as carriers of bioactive compounds or nutrients [44]. Such materials, including leaves, stems, and peels, constitute a largely underexploited resource that can be valorised to improve the nutritional quality of foods while mitigating the environmental burden associated with food waste. This is particularly important, as fruit and vegetable residues, if not appropriately managed, can contribute to soil and water contamination, greenhouse gas emissions, and potential health hazards. These problems are attributed to the high biodegradability of wastes, which is due to their high moisture content and microbial load [32,45,46].

Reducing and valorising food waste and by-products have been widely recommended as efficient strategies to maximise the benefits of available resources and improve food security, sustainability, and circularity in food systems [36,47].

In this context, the utilisation of plant waste materials may provide multiple advantages, encompassing both economic and nutritional benefits. The thiamine fortification of waste vegetable parts may constitute an attractive alternative source of thiamine for all consumers, especially vegetarians and vegans.

Thiamine has been considered the most thermally unstable of the vitamins used in food enrichment [48]. Thiamine is a molecule composed of a pyrimidine ring and a thiazole ring, joined by a methylene bridge. Specifically, it has a 4-amino-2-methylpyrimidine group linked to a 5-(2-hydroxyethyl)-4-methylthiazolium group. According to its structure, thiamine exhibits high biological activity, serving as an enzyme cofactor and having both direct and indirect effects on cell metabolism. The high activity of the thiazole ring causes this vitamin to be highly unstable during food technological processes, including fortification with this vitamin [49]. The special electronic properties of the thiazole ring enable thiamine to act as a crucial electron carrier and intermediate in vital metabolic processes, accounting for its high chemical activity.

The analysis of thiamine degradation kinetics in various matrices has reported that thiamine generally has an activation energy of 20–30 kcal/mol [50,51]. Thiamine is one of the most unstable vitamins due to its sensitivity to temperature variability, and processing and storage conditions [52,53].

Therefore, the effectiveness of thiamine fortification can be influenced by the choice of matrix, highlighting the need to investigate the potential of these materials as carriers for this essential vitamin. The suitability of the product as a matrix for thiamine enrichment is also determined by factors that influence the stability of thiamine in the vitamin-matrix system [54,55]. To the best of our knowledge, no study has been found in the literature that investigates the thiamine fortification of broccoli, carrots, and cauliflower using their waste materials.

Therefore, the investigation aimed to analyse the potential for using broccoli, carrot, and cauliflower waste materials as matrices for thiamine. The study also included non-waste raw materials of these vegetables (peeled carrots without crowns and the florets of cauliflower and broccoli), which served as a reference for analysing the effectiveness of the analysed plant waste as a thiamine matrix. Previous studies on vegetable fortification with thiamine have focused on a single soaking time (60 min) during the impregnation stage conducted at 4 °C [31]. Therefore, the aim of this study was to determine the effect of two soaking times during impregnation at 4 °C (20 and 60 min) on thiamine stability. The 20 min variant was included to assess whether a shorter soaking time could achieve comparable thiamine retention, potentially reducing processing time and costs.

It was hypothesised that the use of analysed plant waste materials (carrots (crowns, peel), cauliflower, and broccoli (stems, leaves)) as a thiamine matrix allows thiamine stability to be maintained at a similar level as that applied to non-waste raw materials of these vegetables. It was also hypothesised that the type of waste plant materials analysed (vegetables, stems, leaves, crowns, and peel) affects the level of this effectiveness.

## 2. Materials and Methods

### 2.1. Materials

Thiamine pyrophosphate (TP) and thiamine hydrochloride (TCh) constituted the sources of thiamine (Merck, Darmstadt, Germany). Vegetables, i.e., cauliflower (*Brassica oleracea* var. *botrytis* L.), broccoli (*Brassica oleracea* L.), and carrot (*Daucus carota* L.), were used as a matrix for the thiamine. The vegetable variables (carrots (crowns, peel), cauliflower, and broccoli (stems, leaves)) were used as a matrix for the thiamine. The peeled carrots (without crowns) and the florets of cauliflower and broccoli were used as reference material to differentiate from the waste fractions (crowns, peel, stems, leaves). These reference parts are included in the table in the last rows. The products were purchased in a ripe state through retail trade in July and August. The vegetables contained thiamine in the range from 0.015 to 0.020 mg/100 g (Table 1).

### 2.2. Methods

#### 2.2.1. Carriers Preparation and Rehydration

The vegetables were washed under running tap water. All the vegetables were separated into anatomical parts: carrots (crowns, peel, peeled carrots without crowns), cauliflower, and broccoli (stems, leaves, florets).

Then they were cut into small pieces. Next, the vegetables were steamed until they were soft (100 °C; 5 min for broccoli and cauliflower, and 8 min for carrots) in a convection oven (Rational, Landsberg am Lech, Germany). The vegetables were subsequently drained and subjected to homogenization (homogeniser—Foss, Hillerød, Denmark). The vegetable impregnation process consisted of two consecutive substages. In the first, vegetables were soaked and mixed in an aqueous solution of thiamine hydrochloride (0.094 mg TCh L^−1^) or thiamine pyrophosphate (0.119 mg TP L^−1^) (total thiamine concentration: 0.07 mg L^−1^) for 10 min at 21 °C. In the second stage, the hydrated vegetables were maintained at 4 °C for either 20 or 60 min, which constituted the main impregnation stage and the variable investigated in this study. After impregnation, the samples were stored at −76 °C for 10 h. Then, the impregnated preparations were freeze-dried (Alpha 1–4 443 LSC Freeze dryer; Christ, Hagen, Germany) at a condenser temperature of −54 °C and a vacuum of 0.520 mbar to achieve a moisture content of 4–5%. The dried vegetables were subjected to homogenisation (homogeniser—Foss, Hillerød, Denmark), to obtain a powder particle size of approximately 250 μm. On average, approximately 9.1 kg of broccoli florets, 7.2 kg of broccoli stems, 5.8 kg of broccoli leaves, 9.8 kg of cauliflower florets, 8.0 kg of cauliflower stems, 5.9 kg of cauliflower leaves, 8.4 kg of peeled carrots, 5.0 kg of carrot peel, and 5.3 kg of carrot leaves were used to produce 1 kg of freeze-dried material.

#### 2.2.2. Storage Conditions of Thiamine Sources

The thiamine-impregnated and freeze-dried vegetables under investigation were stored in jars (black glass, closed with screw top, d = 7 cm, h = 10 cm). The influence of storage conditions on the stability of thiamine was tested during storage at 4, 21, and 40 ± 1 °C.

The thiamine contents in the investigated carriers were monitored on the selected storage days: 0 (immediately after drying), 45, 90, 135, 180, and 230.

#### 2.2.3. Stability of Thiamine

On the set days: before freeze-drying (refers to a sample of the vegetable mixed with a thiamine solution and impregnated for 20 or 60 min, but before it is frozen (a necessary step for freeze-drying)), after drying, and during storage (45, 90, 135, 180, and 230 days), samples were tested for thiamine content using the thiochrome method, which includes the analysis of quantitative changes in the free (thiamine hydrochloride) and bound (thiamine pyrophosphate) form.

The total thiamine content was determined using a modified thiochrome method. A 10 g sample was mixed with 200 mL of 0.1 mol L^−1^ HCl and hydrolysed for 30 min at 95–100 °C with continuous shaking in a rotary shaker. After cooling, pH was adjusted to 4.0–4.5 with 2 mol L^−1^ sodium acetate, and 5 mL of 10% Diastase solution (Merck, Germany) was added. The mixture was incubated for 2.5 h at 45 °C in a shaking water bath, cooled, diluted to 250 mL, and filtered. The filtrate was purified using Amberlite IRC–50 ion-exchange columns as described by Rettenmaier et al. [56]. Thiamine was analysed as its thiochrome derivative following AOAC [57]. For measurement of thiochromium fluorescence, a Jenway model 6200 fluorometer (Jenway, Stone, UK) was used, and measured with a Jenway 6200 fluorometer (365 nm excitation, 435 nm emission). Thiamine content was calculated from a calibration curve (0.1–0.5 μg mL^−1^), with method precision verified using thiamine hydrochloride and thiamine pyrophosphate standards (recoveries of 95% and 92%, respectively). All measurements were performed in triplicate.

Thiamine content was converted to dry weight. For this purpose, the dry mass (DM) of thiamine carriers was estimated by drying at 105 °C to constant weight [58].

### 2.3. Statistical Analysis

The results were analysed statistically with the STATISTICATM PL 13.3 software (StatSoft, Tulsa, OK, USA). The thiamine contents were analysed in 15 samples (three independent samples and five measurements for each sample). The results were subjected to the one-way analysis of variance and Tukey’s test. Hypotheses were tested at α = 0.05. The thiamine data were submitted to linear regression analysis, and the goodness of fitting was evaluated based on statistical parameters of fitting (*R*^2^ and a probability level of the models).

To predict the dynamics of changes in thiamine content in thiamine carriers during storage, losses of 25% (T_25%_) were used. This term refers to the time required for the initial thiamine content to decrease by 25%. The linear reaction of thiamine transformation was taken into account, and the accuracy of the models was estimated using the coefficient of determination (*R*^2^) and root mean square error (*RMSE*). The significance level for all analyses was set at 5% [31].

For the overall evaluation of differences and similarities of the tested samples, the analysis of main components (PCA—Principal Component Analysis) was used.

## 3. Results and Discussion

### 3.1. Thiamine Content After Fortification

**Figure 1:**
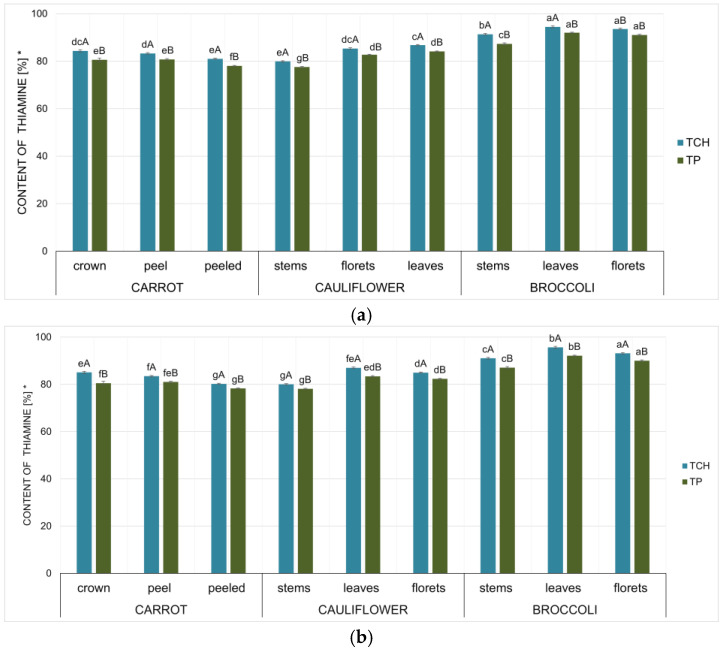
Thiamine content [%] after drying in selected thiamine-fortified vegetables variant with thiamine hydrochloride (TCh) or thiamine pyrophosphate (TP) with the time of impregnation of 20 min (**a**) and 60 min (**b**). Mean values (*n* = 6); different letters (lower case letters in the same thiamine form variant; uppercase letters in the same waste vegetable variant) denote a significant difference at *p* < 0.05 (one-way ANOVA, and post hoc Tukey test). * compared to the content in the product before drying, for the thiamine content converted to dry matter content.

The results of the analysis confirmed the effectiveness of using analysed waste vegetables as a matrix for thiamine application. Figure 1 shows the thiamine content (%) in enriched anatomical parts of vegetables (carrot, broccoli, and cauliflower) after the drying process of samples fortified with thiamine using variable time parameters of the impregnation process (soaking in a thiamine solution for 20 and 60 min). Thiamine content results were converted to dry matter content and presented as a percentage relative to the content in the product before drying (i.e., before freezing the product).

An analysis of thiamine content (Figure 1) revealed a recovery of the introduced thiamine in the product after drying, averaging 88%, compared to the initial content in the fortified vegetables.

The differences in thiamine content ranged from 78% to 98%, compared to the level found in samples immediately after drying. This level is comparable to those reported in other studies and even higher for certain variables [31]. Previous data on the fortification of vegetables (pumpkin, broccoli, cauliflower) confirm the maximum reproducibility of thiamine in fortified matrices at a 75–89% level. These analyses confirmed that the highest effectiveness of thiamine fortification was observed in muscat pumpkin samples (89%), while the lowest effectiveness was found in cauliflower. Thiamine losses during drying are not solely due to transfer to water [59,60]. Thiamine is a very thermally unstable vitamin and sensitive to oxidation processes. Thiamine losses result mainly from the breakdown of the thiazole ring to biologically inactive forms.

Therefore, due to the very low stability of thiamine [49,61,62], the obtained thiamine contents in the dried samples can be considered high. Thiamine is a vitamin that is particularly sensitive to changes in temperature conditions. Thiamine is also susceptible to oxidation. The earlier study confirmed that when stored at 23 °C, mainly thiamine oxidation products were detected, such as thiochrome [62]. Furthermore, the presence of water has been shown to negatively impact the stability of thiamine in the solid state, accelerating the rate of changes, especially oxidative ones [62,63].

The presence of water has been shown to negatively impact the stability of thiamine in the solid state, with degradation rates increasing as relative humidity or aw increase, especially when the deliquescence point is exceeded [63,64]. Therefore, the influence of variable humidity conditions will be addressed in future studies.

The statistical analysis (One-Way ANOVA test) (Table 2) confirmed the strongest relationship between the type of vegetable (F = 50.64; *p* < 0.05) and the thiamine content after drying. The analysis of the effect of the type of vegetable as the matrix for thiamine confirmed that the highest content of thiamine was found in the broccoli samples (87.0–94.4%). A lower thiamine content was confirmed for the samples of cauliflower (79.9–86.8%) and carrots (70.0–84.3%).

The statistical analysis (One-Way ANOVA test) (Table 2) also confirmed a higher relationship between the anatomical part of the vegetable (F = 34.95; *p* < 0.05) and the thiamine content after drying.

Analysis of the waste material type revealed the highest stability of thiamine in leaves, both broccoli and cauliflower. It was confirmed that the thiamine content in the leaves of broccoli ranged from 92.13% to 95.67%, and in cauliflower, it ranged from 83.4% to 87.1%. While in florets of broccoli the thiamine content was 90.0–93.5%, in the florets of cauliflower it ranged from 82.3 to 85.0%. However, for broccoli stems, a lower thiamine recovery was confirmed, at the level of 87.1–91.3%. For the cauliflower stem samples, an even lower thiamine content was confirmed, at the level of 77.5–80.0%. For the samples of carrots, higher thiamine content was confirmed in the fortified crown (80.5–85.0%). These contents were 2 to 6% higher than in peeled carrot samples. The statistical analysis confirmed the lowest relationship between the thiamine form (F = 3.02; *p* = 0.04) and the thiamine content after drying. For all variables, lower thiamine contents in the form of thiamine pyrophosphate were confirmed, averaging 3–4% (Figure 1). Previous data also confirm the lower stability of thiamine in the pyrophosphate form. However, these differences are lower in the impregnated dried products. This can be explained by the fact that less activation energy is required to break the thiazole ring of thiamine [65].

The statistical analysis (One-Way ANOVA test) (Table 2) (*p* = 0.99) did not confirm a relationship between the time of impregnation and the thiamine content after drying. Similar thiamine contents were confirmed for samples impregnated for both 20 min and 60 min. This applies to all tested vegetables and their anatomical parts.

Our previous studies on vegetable fortification with thiamine have focused only on a 60 min time point. To the best of our knowledge, no study has been found in the literature that investigates the thiamine fortification of vegetables using various impregnation times. Previous data confirmed a statistically significant effect of vegetable soaking time in solution on active ingredient losses. However, this was for iodine. The impregnation times in this research were also longer, and, most importantly, iodine has a different chemical nature than thiamine [66].

### 3.2. Thiamine Stability During Storage of Dried Vegetables Fortified with Thiamine

The results of the analysis confirmed the high effectiveness of using analysed waste vegetables as a matrix for thiamine application, based on high thiamine content in the samples after drying. However, the effectiveness of matrix selection for an active ingredient, especially vitamins, is assessed based on its stability during storage. This is particularly true for storage under variable temperature conditions [31,67,68]. Therefore, the study aimed to evaluate the stability of thiamine applied to the tested matrices during storage at 4, 21, and 40 °C. Numerous studies indicate that storing thiamine at 4 °C allows for its high stability [68,69]. However, storing at this temperature is significantly more expensive and requires additional space for refrigeration equipment. The study also included storage variants of pure thiamine hydrochloride or thiamine pyrophosphate samples. This variant was used to evaluate the effectiveness of the tested [31] vegetable matrices for thiamine. The tables containing all the thiamine concentration data are included in Supplementary Materials Tables S1 and S2.

**Figure 2:**
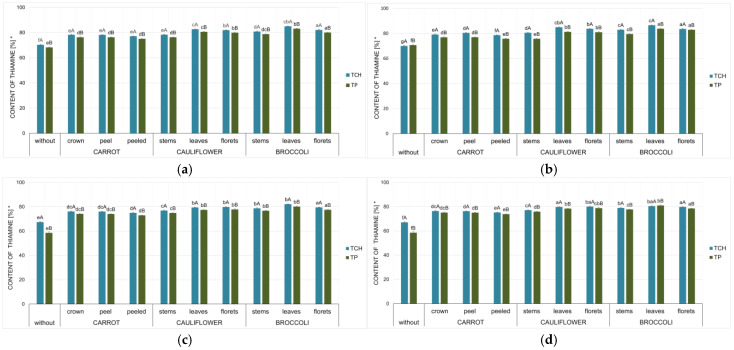

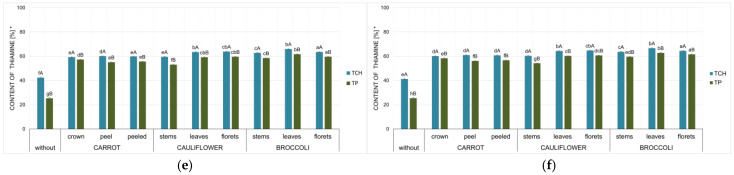
Thiamine content after storage for 230 days [%] compared those immediately after drying in selected thiamine-fortified vegetables with thiamine hydrochloride or thiamine pyrophosphate with the time of impregnation of 20 min (**a**,**c**,**e**) and 60 min (**b**,**d**,**f**), and temperature of storage at 4 °C (**a**,**b**), 21 °C (**c**,**d**), and 40 °C (**e**,**f**). Mean values (*n* = 6); different letters (lowercase letters in the same thiamine form variant; uppercase letters in the same waste vegetable variant) denote a significant difference at *p* < 0.05 (one-way ANOVA, and post hoc Tukey test). * compared to the content in the product before drying, for the thiamine content converted to dry matter content.

Analysis of thiamine content in samples stored for 230 days revealed thiamine levels ranging from 25% to 87% compared to those immediately after drying (Figure 2).

The statistical analysis (One-Way ANOVA test) (Table 3) confirmed the strongest relationship between the storage temperature (F = 4384.00; *p* < 0.05) and thiamine stability during storage.

Analysis of the dynamics of changes in thiamine content (T_25%_) (Table 4 and Table 5) during storage revealed this relationship for all thiamine carriers. It was proved that these alterations occurred in accordance with the first-order reaction kinetics (Table 4). It was found that the lower the storage temperature, the lower the dynamics of thiamine loss. The earlier study confirmed that thiamine-’s degradation during the thermal processing and storage of foods follows first-order kinetics, with associated activation energies ranging from 33 to 124 kJ/mol [53,69,70]. This indicates thiamine’s varying sensitivity to changing environmental conditions. This range varies because food matrices differ in water activity, presence of reducing sugars, oxygen exposure, and pH. The temperature dependence of the rate constant can be described by the Arrhenius equation [57,58]. The statistical analysis (T_25%_) (Table 4, Table 5, Tables S1 and S2) confirmed the fastest rate at 40 °C. The lowest thiamine losses were confirmed for samples stored at 4 °C. The dynamics of thiamine losses were slower for samples stored at 4 °C, in the range of 67–130%, and at 21 °C, in the range of 47–65%, compared to the dynamics of changes in thiamine content at 40 °C. For example, the time of thiamine losses at the level of 25% for the preparation samples of broccoli leaves enriched with thiamine hydrochloride was 183–188 days at 40 °C, and for samples stored at 4 °C, 392–435 days. For samples stored at 21 °C, the time of 25% thiamine loss was from 206 to 335 days.

Previous studies have also confirmed the higher instability of thiamine to both high-temperature processing and storage [71]. Analysis of mixtures containing nutrients revealed a significantly faster rate of thiamine metabolism, leading to increased thiamine losses with increasing storage temperature [72]. Another study of enriched pasta confirmed that the rate constant for thiamine loss was higher at 45 °C than at 25 °C or 35 °C on 20–30% [69]. It was also confirmed for thermal degradation kinetics of ascorbic acid, thiamine, and riboflavin in rosehip (*Rosa canina* L.) nectar [73]. This can be explained by a smaller amount of activation energy required to break down the thiazole ring of thiamine [74].

Statistical analysis confirmed that thiamine degradation was highest in samples stored at 40 °C. However, this process was significantly slowed when thiamine was applied to vegetable matrices. Regardless of storage temperature, the lowest thiamine retention was consistently observed in samples without a matrix, in which thiamine was added solely as pure thiamine hydrochloride or thiamine pyrophosphate. These trends were evident in all analysed storage temperature variants. At 40 °C, thiamine retention was 25–42% in samples without a vegetable matrix and 54–66% in vegetable-based samples. After 230 days at 4 °C, retention was 68–70% and 75–86% in samples without and with a vegetable matrix, respectively.

It was also confirmed that thiamine stability was influenced by thiamine form (F = 8.65; *p* < 0.05), with consistently lower stability observed for thiamine pyrophosphate.

The statistical analysis of predictors in variance models for changes in thiamine content in the analysed vegetable samples after storage (Table 3) confirmed that thiamine stability was significantly influenced by the type of vegetable (F = 13.85; *p* < 0.05). The highest thiamine retention after 230 days was observed in broccoli samples, which was 47% higher than in pure thiamine samples (Figure 2), while the lowest retention was found in carrot samples; however, even in these samples, it was still 37% higher than in samples without a vegetable matrix (Figure 2).

The thiamine stability also depended on the anatomical part of the vegetable (F = 6.66; *p* < 0.05), namely, leaves, florets, or stems. The statistical analysis of broccoli and cauliflower waste confirmed that the half-life of thiamine was longest for the leaf samples (Table 4 and Table 5). The half-life was up to 20% higher in broccoli leaves and up to 12% higher in cauliflower leaves compared with florets.

**Figure 3:**
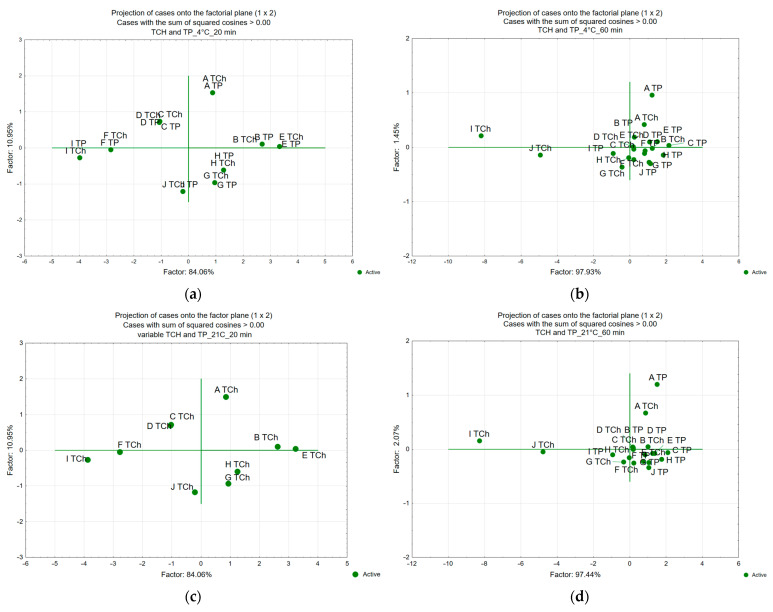

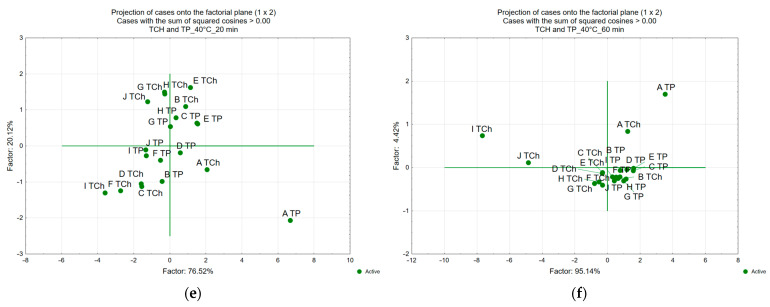
A map of the variants of thiamine content of waste plants (carrots (crowns, peel), cauliflower, and broccoli (stems, leaves)) and peeled carrots, without crowns, florets of cauliflower, and broccoli fortified with thiamine hydrochloride (TCh) and thiamine pyrophosphate (TP) with the various time of impregnation of 20 min (**a**,**c**,**e**) and 60 min (**b**,**d**,**f**), and temperature of storage at 4 °C (**a**,**b**), 21 °C (**c**,**d**), and 40 °C (**e**,**f**) (F1 × F2). Case-factor coordinate plots based on the attributes of taste profiles (PCA analysis); with Letter designations: A TCh (Ch without vegetable), B TCh (TCh crown of carrot); C TCh (TCh Cheel of carrot); D TCh (TCh Cheeled carrot); E TCh (TCh stems of cauliflower); F TCh (TCh florets of cauliflower); G TCh (TCh leaves of cauliflower); H TCh (TCh stems of broccoli); I TCh (TCh florets of broccoli); J TCh (TCh leaves of broccoli); A TP (TP without vegetable); B TP (TP crown of carrot); C TP (TP peel of carrot); D TP (TP peeled carrot); E TP (TP stems of cauliflower); F TP (TP florets of cauliflower); G TP (TP leaves of cauliflower); H TP (TP stems of broccoli); I TP (TP florets of broccoli); J TP (TP leaves of broccoli); (F1 × F2): Case-factor coordinate plots based on taste profiles (PCA analysis) after 230 days of storage.

Using broccoli leaves as a matrix provided the highest thiamine stability among the matrices tested. Thiamine content in leaves ranged from 62% to 87%, and was lower in florets (60–83%) and lowest in stems (58–82%) after 230 days of storage. In cauliflower, thiamine content after 230 days ranged from 53% to 83%. It was lowest in impregnated stem samples (53–80%), whereas leaves and florets showed similar values (59–83%). The smallest differences among anatomical parts were observed in carrot samples (Table 3; Figure 3). Thiamine stability analysis indicated that the carrot crown was the most effective matrix. Its thiamine content was, on average, 2–4% higher than in both carrot peel and peeled carrot samples. The content in carrot peel was generally similar to or slightly higher (by up to 2%) than in peeled carrot. The variable stability of thiamine in the vegetable samples studied may be related to their composition. Previous data also confirm the high stability of thiamine applied to broccoli and lower stability to carrots [31].

Previous studies have confirmed potential interactions between thiamine and polyphenols, as well as caffeine. Theoretical studies on bond dissociation energy (BDE) have also demonstrated the possibility of complex formation between thiamine and epigallocatechin gallate [75,76]. Studies confirm the high affinity of thiamine for tea flavonoids—(−)-epigallocatechin gallate (EGCG) [77,78,79] and caffeine [80]. Broccoli leaves and stems are often considered waste products. However, they are a valuable source of bioactive substances such as phenolic compounds, glucosinolates, flavonoids, carotenoids, and sterols [73]. According to Garcia and Raghavan [73], broccoli leaves have the highest phenolic compound content, followed by florets and stems. Furthermore, broccoli leaves also exhibit the highest antioxidant activity [73,81], which may contribute to the enhanced thiamine stability observed in these samples, as previous studies have shown that thiamine stability is improved in systems containing active antioxidant compounds, such as epigallocatechin gallate, rosemary extract, and casein hydrolysate, supporting the trend that matrices with higher antioxidant capacity provide greater thiamine retention during processing and storage [80,82]. Among the phenolic compounds identified in broccoli leaves and stems were characterised by high levels of neochlorogenic acid, chlorogenic acid, and sinapic acid. Furthermore, an analysis of the active ingredient content in various anatomical parts of broccoli conducted by Liu et al. [74] revealed that broccoli leaves exhibited higher concentrations of carotenoids, chlorophylls, vitamin E, vitamin K, calcium, and manganese. According to El-Sawi et al. [83], cauliflower leaves also have higher antioxidant activity than the florets or stems of this plant, which is correlated with the content of the above-mentioned bioactive substances. The phenolic compounds found in the highest amounts in cauliflower leaves were hesperidin, catechin, pyrogallol, apigenin-6-O-arabinose-8-O-galactose, naringin, luteolin-7-O-glucoside, and benzoic acid [83]. Previous studies have confirmed the influence of reducing sugars (xylose, glucose, and maltose) on the rate of thiamine destruction. The rate of thiamine degradation depends on both the type and concentration of reducing sugars, with higher sugar levels accelerating vitamin loss during storage. Thiamine participates in reactions with reducing sugars, particularly in non-enzymatic browning (Maillard) reactions, resulting in chemical degradation and loss of nutritional activity [84,85]. Thiamine destruction rates, in the presence of a reducing sugar, decreased in the order: xylose > glucose > maltose [86,87,88]. In addition, for the three reducing sugars studied, thiamine loss was enhanced as reducing sugar concentration increased. It was found that, when a reducing sugar was present, the rate of thiamine loss increased by as much as 37%. The higher sugar content can explain the lower thiamine stability in broccoli stems compared to leaves, as is the case with carrots [89].

Statistical analysis did not confirm the effect of impregnation time (20/60 min) (F = 0.1; *p* = 0.76). Previous studies confirmed the effect of impregnation time on iodine stability. However, the time of impregnation was longer [66]. These studies analysed the impact of impregnation for up to 6 h, and it was confirmed that higher stability of iodine was achieved for the samples with the impregnation at 6 h. Other studies on fortifying apples with vitamin B12 confirmed that extending the impregnation time from 5 to 15 min increased the effectiveness of the process [90]. The lack of differences in thiamine content may be due to higher activity of thiamine in its dissolved form. For samples impregnated for 60 min, thiamine remains dissolved for a more extended period. Thiamine dissolved in water exhibits higher lability [63]. This was also confirmed by the greater variability in the results (Figure 3a–f).

This study focused on the effect of temperature on thiamine stability. The content of phenolic and flavonoid compounds was not quantified, and the effect of storage under varying humidity conditions was not assessed. Therefore, both the variability in bioactive compounds and storage conditions, particularly with respect to variable humidity, should be considered in future studies.

## 4. Conclusions

The analysis confirmed the effectiveness of using carrot, cauliflower, and broccoli waste as a matrix for thiamine.

The recovery of thiamine in the product after drying ranged from 78% to 98% compared to its content before drying.

Thiamine stability was markedly higher when incorporated into vegetable matrices. The lowest thiamine retention was consistently observed in samples without a matrix, where thiamine was present only as pure thiamine hydrochloride or thiamine pyrophosphate. The results confirmed the variable stability of thiamine in the analysed vegetables during storage, as follows in order of decreasing stability: leaves of broccoli > florets of broccoli > leaves of cauliflower > stems of broccoli = florets of cauliflower > peeled carrot = stems of cauliflower = peel of carrot > crown of carrot.

However, for maximum effectiveness in using analysed vegetables, as well as their waste, as a matrix for thiamine application, it is suggested to store them at 4 °C or 21 °C.

The results may be of interest to nutritionists and to food producers who offer food to consumers at risk of thiamine deficiency, such as vegans, vegetarians, young people, and older individuals. Further research should involve adding the designed extracts fortified with thiamine to selected food products, such as flour or meat products.

## Figures and Tables

**Table 1:** Content of thiamine in the vegetable samples before fortification [mg/100 g].

CARROT			CAULIFLOWER			BROCCOLI		
Crown	Peel	Peeled	Stems	Florets	Leaves	Stems	Florets	Leaves
0.011	0.011	0.012	0.012	0.013	0.012	0.018	0.020	0.017

**Table 2:** Statistical significance of predictors of variance models for changes in thiamine content in selected thiamine-fortified vegetables variant after drying (One-Way ANOVA test).

Predictors	SS	df	MSE	F-Value	*p*-Value
Time impregnation	0.00	1.00	0.00	0.00	0.99
Thiamine form	79.60	1.00	79.60	3.02	0.04
Anatomical part vegetable	890.40	8.00	111.30	34.95	0.00
Type vegetable	736.40	2.00	368.20	50.64	0.00

SS—Statistical Significance; df—degrees of freedom; MSE—mean sum of squares.

**Table 3:** Statistical significance of predictors of variance models for changes in thiamine content in selected thiamine-fortified vegetables after storage (230 days) (One-Way ANOVA test).

Predictors	SS	df	MSE	F-Value	*p*-Value
Analysis for all samples (TCh and TP)					
Time impregnation	14.00	1.00	14.00	0.10	0.76
Thiamine form	1303.00	1.00	1303.00	8.65	0.00
Temp. storage	11,933.00	2.00	5966.00	43.84	0.00
Type vegetable	6008.00	3.00	2003.00	13.85	0.00
Anatomical part vegetable	5809.00	6.00	968.00	6.66	0.00
TCh					
Time impregnation	8.50	1.00	8.50	0.08	0.78
Temp. of storage	4577.70	2.00	2288.90	86.41	0.00
Type vegetable	1284.70	3.00	428.20	4.99	0.00
Anatomical part vegetable	1371.30	9.00	152.40	1.62	0.14
TP					
Time impregnation	14.30	1.00	14.30	0.09	0.77
Temp. of storage	6214.10	2.00	3107.10	57.80	0.00
Type vegetable	2386.60	3.00	795.50	6.47	0.00
Anatomical part vegetable	2515.20	9.00	279.50	2.07	0.00
Temperature of storage 4 °C					
Time impregnation	16.20	1.00	16.20	0.89	0.35
Thiamine form	52.80	1.00	52.80	3.06	0.04
Type vegetable	538.30	3.00	179.40	37.98	0.00
Anatomical part vegetable	626.10	9.00	69.60	25.37	0.00
Temperature of storage 21 °C					
Time impregnation	2.00	1.00	2.00	0.07	0.79
Thiamine form	50.40	1.00	50.40	1.97	0.04
Type vegetable	877.10	3.00	292.40	72.19	0.00
Anatomical part vegetable	919.10	9.00	102.10	29.53	0.00
Temperature of storage 40 °C					
Time impregnation	7.60	1.00	7.60	0.09	0.77
Thiamine form	287.80	1.00	287.80	3.71	0.04
Type vegetable	2693.15	3.00	897.72	59.81	0.00
Anatomical part vegetable	2788.00	9.00	309.80	20.86	0.00

SS—Statistical Significance; df—degrees of freedom; MSE—mean sum of squares.

**Table 4:** Dynamics of changes in thiamine content (mg TCh or TP kg^−1^) in carriers (the dried thiamine-fortified vegetables samples (20 min of impregnation)) during 230 days of storage with various temperatures of storage as values of half time of thiamine degradation (T_25%_) and coefficients in regression equations.

Parameters Fortifications		Dynamics of Change in Thiamine Content Over 230 Days									
Vegetable	Anatomical Part	T_25%_ [Days]	*R* ^2^	*RMSE*	Y = ax + b		T_25%_ [Days]	*R* ^2^	*RMSE*	Y = ax + b	
					Coeff. a24 h^−1^	b				Coeff. a24 h^−1^	b
		TCh					TP				
20 min of impregnation 4 °C of storage											
without		211.65	0.967	0.33	−0.0013	3.020	194.93	0.976	0.05	−0.0014	3.080
CARROT	crown of carrot	251.31	0.951	0.13	−0.0010	1.022	225.71	0.944	0.15	−0.0011	2.976
	peel of carrot	254.83	0.954	0.04	−0.0011	1.242	231.08	0.954	0.02	−0.0011	2.828
	peeled carrot	255.17	0.961	0.02	−0.0011	8.864	230.61	0.961	0.02	−0.0011	2.916
CAULIFLOWER	stems of cauliflower	255.57	0.952	0.02	−0.0010	1.022	230.85	0.952	0.02	−0.0011	2.827
	leaves of cauliflower	344.95	0.924	0.06	−0.0007	8.638	307.85	0.936	0.02	−0.0008	2.872
	florets of cauliflower	317.39	0.972	0.11	−0.0009	1.239	281.18	0.969	0.01	−0.0009	2.957
BROCCOLI	stems of broccoli	315.37	0.969	0.02	−0.0008	1.022	284.27	0.987	0.01	−0.0009	2.869
	leaves of broccoli	392.47	0.971	0.06	−0.0007	8.500	355.16	0.988	0.01	−0.0008	2.943
	florets of broccoli	335.73	0.937	0.06	−0.0009	1.239	306.14	0.968	0.01	−0.0009	2.978
20 min of impregnation 21 °C of storage											
without		186.11	0.987	0.307	−0.0016	3.031	149.42	0.984	0.11	−0.0020	3.118
CARROT	crown of carrot	228.43	0.960	0.148	−0.0011	2.801	206.55	0.952	0.17	−0.0012	2.970
	peel of carrot	232.12	0.964	0.057	−0.0012	3.029	210.98	0.962	0.02	−0.0012	2.824
	peeled carrot	232.44	0.970	0.016	−0.0012	3.030	211.22	0.968	0.02	−0.0012	2.911
CAULIFLOWER	stems of cauliflower	237.41	0.954	0.018	−0.0010	2.762	215.28	0.951	0.02	−0.0011	2.817
	leaves of cauliflower	292.68	0.965	0.065	−0.0009	2.818	264.02	0.970	0.01	−0.0010	2.876
	florets of cauliflower	283.34	0.981	0.103	−0.0010	3.066	254.26	0.977	0.01	−0.0010	2.944
BROCCOLI	stems of broccoli	283.31	0.979	0.012	−0.0009	2.834	257.32	0.989	0.01	−0.0010	2.863
	leaves of broccoli	335.87	0.977	0.057	−0.0008	2.866	302.71	0.992	0.01	−0.0009	2.953
	florets of broccoli	296.47	0.950	0.074	−0.0010	3.132	271.46	0.973	0.01	−0.0010	2.976
20 min of impregnation 40 °C of storage											
without		109.29	0.984	0.206	−0.0026	3.015	70.52	0.987	0.21	−0.0036	2.970
CARROT	crown of carrot	144.59	0.992	0.255	−0.0019	2.854	134.67	0.994	0.28	−0.0020	3.031
	peel of carrot	148.61	0.983	0.092	−0.0020	3.084	129.43	0.984	0.02	−0.0021	2.873
	peeled carrot	149.77	0.985	0.019	−0.0019	3.081	131.21	0.986	0.02	−0.0021	2.952
CAULIFLOWER	stems of cauliflower	148.19	0.981	0.020	−0.0018	2.817	128.12	0.978	0.03	−0.0021	2.882
	leaves of cauliflower	171.11	0.978	0.056	−0.0016	2.866	148.84	0.987	0.02	−0.0019	2.920
	florets of cauliflower	166.41	0.989	0.102	−0.0018	3.123	144.21	0.993	0.01	−0.0019	2.993
BROCCOLI	stems of broccoli	168.21	0.972	0.024	−0.0017	2.882	147.11	0.988	0.02	−0.0019	2.906
	leaves of broccoli	183.86	0.970	0.050	−0.0016	2.916	160.29	0.986	0.02	−0.0018	3.014
	florets of broccoli	175.23	0.955	0.175	−0.0018	3.190	155.54	0.976	0.02	−0.0019	3.045

**Table 5:** Dynamics of changes in thiamine content (mg TCh or TP kg^−1^) in carriers (the dried thiamine-fortified vegetables (60 min of impregnation)) during 230 days of storage with various temperatures of storage as values of half time of thiamine degradation (T_25%_) and coefficients in regression equations.

Parameters Fortifications		Dynamics of Change in Thiamine Content Over 230 Days									
Vegetable	Anatomical Part	T_25%_ [Days]	*R* ^2^	*RMSE*	Y = ax + b		T_25%_ [Days]	*R* ^2^	*RMSE*	Y = ax + b	
					Coeff. a24 h^−1^	b				Coeff. a24 h^−1^	b
		TCh					TP				
60 min of impregnation 4 °C of storage											
without		210.36	0.961	0.34	−0.0014	3.200	171.76	0.927	0.08	−0.0017	3.202
CARROT	crown of carrot	268.51	0.963	0.13	−0.0010	2.956	234.23	0.944	0.16	−0.0011	3.221
	peel of carrot	281.83	0.952	0.05	−0.0010	3.165	237.74	0.961	0.02	−0.0010	2.802
	peeled carrot	275.86	0.965	0.02	−0.0010	3.195	238.92	0.961	0.02	−0.0011	3.036
CAULIFLOWER	stems of cauliflower	282.92	0.958	0.02	−0.0009	3.028	232.96	0.958	0.05	−0.0011	2.951
	leaves of cauliflower	396.49	0.930	0.04	−0.0007	3.208	321.06	0.930	0.02	−0.0008	2.932
	florets of cauliflower	354.29	0.981	0.04	−0.0008	3.103	297.08	0.968	0.01	−0.0009	3.015
BROCCOLI	stems of broccoli	350.27	0.961	0.03	−0.0008	3.177	295.35	0.984	0.01	−0.0009	2.813
	leaves of broccoli	435.38	0.956	0.19	−0.0009	4.390	368.34	0.990	0.01	−0.0007	2.901
	florets of broccoli	362.04	0.908	0.40	−0.0012	5.657	346.94	0.982	0.01	−0.0009	3.381
60 min of impregnation 21 °C of storage											
without		185.52	0.985	0.33	−0.0016	3.218	148.36	0.989	0.11	−0.0021	3.239
CARROT	crown of carrot	234.03	0.960	0.15	−0.0011	2.949	214.79	0.948	0.17	−0.0012	3.214
	peel of carrot	237.71	0.964	0.06	−0.0012	3.190	220.11	0.962	0.02	−0.0011	2.793
	peeled carrot	237.66	0.967	0.02	−0.0012	3.192	219.47	0.966	0.02	−0.0012	3.030
CAULIFLOWER	stems of cauliflower	243.34	0.954	0.02	−0.0011	3.027	224.44	0.949	0.04	−0.0011	2.931
	leaves of cauliflower	298.53	0.962	0.04	−0.0010	3.219	276.99	0.967	0.01	−0.0009	2.935
	florets of cauliflower	290.54	0.977	0.04	−0.0010	3.103	266.90	0.975	0.01	−0.0010	3.005
BROCCOLI	stems of broccoli	289.46	0.972	0.02	−0.0010	3.174	269.93	0.989	0.01	−0.0010	2.807
	leaves of broccoli	309.01	0.963	0.20	−0.0012	4.412	317.43	0.986	0.01	−0.0008	2.902
	florets of broccoli	301.85	0.938	0.37	−0.0015	5.674	285.16	0.974	0.01	−0.0011	3.390
60 min of impregnation 40 °C of storage											
without		107.21	0.983	0.21	−0.0028	3.203	69.83	0.985	0.21	−0.0036	2.970
CARROT	crown of carrot	148.59	0.992	0.26	−0.0019	3.007	139.80	0.994	0.28	−0.0020	3.031
	peel of carrot	152.71	0.983	0.10	−0.0020	3.250	134.21	0.982	0.02	−0.0021	2.873
	peeled carrot	154.04	0.985	0.02	−0.0020	3.246	136.23	0.985	0.02	−0.0021	2.952
CAULIFLOWER	stems of cauliflower	152.26	0.981	0.02	−0.0019	3.091	132.69	0.977	0.03	−0.0021	2.882
	leaves of cauliflower	176.29	0.976	0.04	−0.0018	3.279	154.65	0.984	0.02	−0.0019	2.920
	florets of cauliflower	171.38	0.989	0.05	−0.0018	3.160	149.93	0.991	0.01	−0.0019	2.993
BROCCOLI	stems of broccoli	172.94	0.969	0.03	−0.0018	3.233	152.65	0.984	0.02	−0.0019	2.906
	leaves of broccoli	188.89	0.957	0.16	−0.0022	4.523	166.11	0.983	0.02	−0.0019	3.049
	florets of broccoli	179.89	0.958	0.28	−0.0026	5.809	161.45	0.983	0.02	−0.0019	3.045

## Data Availability

The data presented in this study are available on request from the corresponding author.

## Supplementary Materials

The following supporting information can be downloaded at: https://www.mdpi.com/article/10.3390/foods15050801/s1, Table S1: The thiamine content (%) during 230 days of storage of the dried thiamine hydrochloride fortified vegetables with various times of impregnation (20 and 60 min); Table S2: The thiamine content (%) during 230 days of storage of the dried thiamine pyrophosphate fortified vegetables with various times of impregnation (20 and 60 min).

## References

[1] World Health Organization (2023). Carbohydrate Intake for Adults and Children WHO Guideline.

[2] Afshin A., Sur P.J., Fay K.A., Cornaby L., Ferrara G., Salama J.S., Mullany E.C., Abate K.H., Abbafati C., Abebe Z. (2019). Health Effects of Dietary Risks in 195 Countries, 1990–2017: A Systematic Analysis for the Global Burden of Disease Study 2017. Lancet.

[3] Ahmadzadeh S., Clary T., Rosales A., Ubeyitogullari A. (2024). Upcycling Imperfect Broccoli and Carrots into Healthy Snacks Using an Innovative 3D Food Printing Approach. Food Sci. Nutr..

[4] Dias J.S. (2012). Nutritional Quality and Health Benefits of Vegetables: A Review. Food Nutr. Sci..

[5] Stea T.H., Nordheim O., Bere E., Stornes P., Eikemo T.A. (2020). Fruit and Vegetable Consumption in Europe According to Gender, Educational Attainment and Regional Affiliation—A Cross-Sectional Study in 21 European Countries. PLoS ONE.

[6] Wallace T.C., Bailey R.L., Blumberg J.B., Burton-Freeman B., Chen C.O., Crowe-White K.M., Drewnowski A., Hooshmand S., Johnson E., Lewis R. (2020). Fruits, Vegetables, and Health: A Comprehensive Narrative, Umbrella Review of the Science and Recommendations for Enhanced Public Policy to Improve Intake. Crit. Rev. Food Sci. Nutr..

[7] de Escalada Pla M.F., Flores S.K., Genevois C.E. (2020). Innovative Strategies and Nutritional Perspectives for Fortifying Pumpkin Tissue and Other Vegetable Matrices with Iron. Food Sci. Hum. Wellness.

[8] Liubych V., Novikov V., Pushka O., Pushka I., Cherchel V., Kyrpa M., Kolibabchuk T., Kirian V., Moskalets V., Moskalets T. (2023). Development of the Recipe of Pasta with Pumpkin Flour. EUREKA Life Sci..

[9] de Valença A.W., Bake A., Brouwer I.D., Giller K.E. (2017). Agronomic Biofortification of Crops to Fight Hidden Hunger in Sub-Saharan Africa. Glob. Food Secur..

[10] Li Y.O., Komarek A.R. (2017). Dietary Fibre Basics: Health, Nutrition, Analysis, and Applications. Food Qual. Saf..

[11] Barber T.M., Kabisch S., Pfeiffer A.F.H., Weickert M.O. (2020). The Health Benefits of Dietary Fibre. Nutrients.

[12] Deehan E.C., Mocanu V., Madsen K.L. (2024). Effects of Dietary Fibre on Metabolic Health and Obesity. Nat. Rev. Gastroenterol. Hepatol..

[13] Oliviero T., Fogliano V. (2016). Food Design Strategies to Increase Vegetable Intake: The Case of Vegetable Enriched Pasta. Trends Food Sci. Technol..

[14] Smith T.J., Johnson C.R., Koshy R., Hess S.Y., Qureshi U.A., Mynak M.L., Fischer P.R. (2021). Thiamine Deficiency Disorders: A Clinical Perspective. Ann. N. Y. Acad. Sci..

[15] Gomes F., Bergeron G., Bourassa M.W., Fischer P.R. (2021). Thiamine Deficiency Unrelated to Alcohol Consumption in High-Income Countries: A Literature Review. Ann. N. Y. Acad. Sci..

[16] Marrs C., Lonsdale D. (2021). Hiding in Plain Sight: Modern Thiamine Deficiency. Cells.

[17] Whitfield K.C., Smith G., Chamnan C., Karakochuk C.D., Sophonneary P., Kuong K., Dijkhuizen M.A., Hong R., Berger J., Green T.J. (2017). High Prevalence of Thiamine (Vitamin B1) Deficiency in Early Childhood among a Nationally Representative Sample of Cambodian Women of Childbearing Age and Their Children. PLoS Negl. Trop. Dis..

[18] Mallimoggala Y.S.P., Biswas M., Anburaj S.E., Iqbal F., Shrikiran A., Suryakanth V.B., Lewis L.E.S. (2024). Thiamine: An Indispensable Regulator of Paediatric Neuro-Cardiovascular Health and Diseases. Eur. J. Pediatr..

[19] Zhuo S., Chen Z., Ye L., Chen J., Yu Z., Yang M. (2025). Association between Dietary Vitamin B1 Intake and Stroke Risk in Older Patients: A Retrospective Cross-Sectional Study. BMC Neurol..

[20] Gibson G.E., Hirsch J.A., Fonzetti P., Jordan B.D., Cirio R.T., Elder J. (2016). Vitamin B1 (Thiamine) and Dementia. Ann. N. Y. Acad. Sci..

[21] Uchida N., Ishida M., Sato I., Yoshioka A., Takahashi T., Furuya D., Ebihara Y., Ito H., Onishi H. (2023). The Prevalence of Thiamine Deficiency among Elderly Nursing Home Residents: A Cross-Sectional Study. J. Gen. Fam. Med..

[22] Nisar S., Yousuf wani I., Altaf U., Muzaffer U., Kareem O., Tanvir M., Ganie M.A. (2024). Thiamine Deficiency-Related Neuropathy: A Reversible Entity from an Endemic Area. Eur. J. Neurol..

[23] Alvarez M., Poveda S., Cisneros A., Parra D., Luna M., Rincón O., Guzman I. (2026). B Vitamin Deficiencies and Associated Neuropathies. Curr. Nutr. Rep..

[24] Osiezagha K., Ali S., Freeman C., Barker N.C., Jabeen S., Maitra S., Olagbemiro Y., Richie W., Bailey R.K. (2013). Thiamine Deficiency and Delirium. Innov. Clin. Neurosci..

[25] Hung S.C., Hung S.H., Tarng D.C., Yang W.C., Chen T.W., Huang T.P. (2001). Thiamine Deficiency and Unexplained Encephalopathy in Hemodialysis and Peritoneal Dialysis Patients. Am. J. Kidney Dis..

[26] Dhir S., Tarasenko M., Napoli E., Giulivi C. (2019). Neurological, Psychiatric, and Biochemical Aspects of Thiamine Deficiency in Children and Adults. Front. Psychiatry.

[27] Stawny M., Gostyńska A., Olijarczyk R., Jelińska A., Ogrodowczyk M. (2020). Stability of High-Dose Thiamine in Parenteral Nutrition for Treatment of Patients with Wernicke’s Encephalopathy. Clin. Nutr..

[28] Wilson R.B. (2020). Pathophysiology, Prevention, and Treatment of Beriberi after Gastric Surgery. Nutr. Rev..

[29] Green T.J., Whitfield K.C., Daniels L., Brown R.C., Houghton L.A. (2021). Modeling Thiamine Fortification: A Case Study from Kuria Atoll, Republic of Kiribati. Ann. N. Y. Acad. Sci..

[30] Mrowicka M., Mrowicki J., Dragan G., Majsterek I. (2023). The Importance of Thiamine (Vitamin B1) in Humans. Biosci. Rep..

[31] Szymandera-Buszka K., Piechocka J., Zaremba A., Przeor M., Jędrusek-Golińska A. (2021). Pumpkin, Cauliflower and Broccoli as New Carriers of Thiamine Compounds for Food Fortification. Foods.

[32] Nartea A., Fanesi B., Pacetti D., Lenti L., Fiorini D., Lucci P., Frega N.G., Falcone P.M. (2023). Cauliflower By-Products as Functional Ingredient in Bakery Foods: Fortification of Pizza with Glucosinolates, Carotenoids and Phytosterols. Curr. Res. Food Sci..

[33] Fartoosi Z., Heidarizadeh F., Kolahi M., Goldson-Barnaby A. (2025). Phytochemical and Nutritional Analysis of *Daucus carota* L. (Carrots) Exposed to Various Processing Conditions. Appl. Food Res..

[34] Motegaonkar S., Shankar A., Tazeen H., Gunjal M., Payyanad S. (2024). A Comprehensive Review on Carrot (*Daucus carota* L.): The Effect of Different Drying Methods on Nutritional Properties and Its Processing as Value-Added Foods †. Sustain. Food Technol..

[35] Andrés C.M.C., Pérez de la Lastra J.M., Munguira E.B., Juan C.A., Pérez-Lebeña E. (2025). The Multifaceted Health Benefits of Broccoli—A Review of Glucosinolates, Phenolics and Antimicrobial Peptides. Molecules.

[36] Aït-Kaddour A., Hassoun A., Tarchi I., Loudiyi M., Boukria O., Cahyana Y., Ozogul F., Khwaldia K. (2024). Transforming Plant-Based Waste and by-Products into Valuable Products Using Various “Food Industry 4.0” Enabling Technologies: A Literature Review. Sci. Total Environ..

[37] Shinali T.S., Zhang Y., Altaf M., Nsabiyeze A., Han Z., Shi S., Shang N. (2024). The Valorization of Wastes and Byproducts from Cruciferous Vegetables: A Review on the Potential Utilization of Cabbage, Cauliflower, and Broccoli Byproducts. Foods.

[38] Pietrangeli R., Cicatiello C. (2024). Lost Vegetables, Lost Value: Assessment of Carrot Downgrading and Losses at a Large Producer Organisation. J. Clean. Prod..

[39] Mirabella N., Castellani V., Sala S. (2014). Current Options for the Valorization of Food Manufacturing Waste: A Review. J. Clean. Prod..

[40] Sahu S., Balakrishnan M., Ramalakshmi A., Rhim J.W., Sahu T. (2025). New Sources, Stability, and Bioavailability for Micronutrient Fortification of 3D-Printed Foods. Trends Food Sci. Technol..

[41] Esparza I., Jiménez-Moreno N., Bimbela F., Ancín-Azpilicueta C., Gandía L.M. (2020). Fruit and Vegetable Waste Management: Conventional and Emerging Approaches. J. Environ. Manag..

[42] Kumar S., Aalbersberg B. (2006). Nutrient Retention in Foods after Earth-Oven Cooking Compared to Other Forms of Domestic Cooking{star, Open}{star, Open}Results of This Work Are Printed as a Technical Report with Limited Distribution by the Institute of Applied Sciences at The University. J. Food Compos. Anal..

[43] Sagar N.A., Pareek S., Sharma S., Yahia E.M., Lobo M.G. (2018). Fruit and Vegetable Waste: Bioactive Compounds, Their Extraction, and Possible Utilization. Compr. Rev. Food Sci. Food Saf..

[44] Francioso O. (2024). Current and Future Perspectives for Biomass Waste Management and Utilization. Sci. Rep..

[45] Taheri S., Hosseini S.S. (2025). Waste Not, Want Not: Comprehensive Valorization of Fruit and Vegetable Waste from Single-Product Recovery to Zero-Waste Strategies. Clean. Waste Syst..

[46] Bain M., Soligo D., van der Werf P., Parizeau K. (2024). The Limitations of an Informational Campaign to Reduce Household Food Waste at the Community Scale. Clean. Waste Syst..

[47] McClements I.F., McClements D.J. (2023). Toward Designing Healthier Plant-Based Foods: Fortification, Digestion. Food Res. Int..

[48] Labuza T.P., Kamman J.F. (1982). Comparison of Stability of Thiamin Salts at High Temperature and Water Activity. J. Food Sci..

[49] Mauer L.J., Dwivedi B.K., Arnold R.G. (1972). Chemistry of Thiamine Degradation: 4-Methyl-S(β-Hydroksyethyl)Thiazole from Thermally Degradation Thiamine. J. Food Sci..

[50] Kamman J.F., Labuza T.P., Warthesen J.J. (1981). Kinetics of Thiamin and Riboflavin Loss in Pasta as a Function of Constant and Variable Storage Conditions. J. Food Sci..

[51] Mauri L.M., Alzamora S.M., Tomio J.M. (1992). Effect of Electrolytes on the Kinetics of Thiamine Loss in Model Systems of High Water Activity. Food Chem..

[52] Ramaswamy H., Ghazala S., van de Voort F. (1990). Degradation Kinetics of Thiamine in Aqueous Systems at High Temperatures. Can. Inst. Food Sci. Technol. J..

[53] Yang H., Xu L.L., Hou L., Xu T.C., Ye S.H. (2022). Stability of Vitamin A, E, C and Thiamine during Storage of Different Powdered Enteral Formulas. Heliyon.

[54] Habib P., Dang J., Slowik A., Victor M., Beyer C. (2014). Hypoxia-Induced Gene Expression of Aquaporin-4, Cyclooxygenase-2 and Hypoxia-Inducible Factor 1α in Rat Cortical Astroglia Is Inhibited by 17β-Estradiol and Progesterone. Neuroendocrinology.

[55] Huang J.H. (2022). Effect of Metal Ions and Temperature on Stability of Thiamine Effect of Metal Ions and Temperature on Stability of Thiamine Determined by HPLC Determined by HPLC. All Theses.

[56] Rettenmaier R., Vuilleumier J.P., Müller-Mulot W. (1979). Zur Quantitativen Vitamin-B1- Bestimmung in Nahrungsmitteln Und Biolgischem Material. Lebensm. Unteres. Forsch. Ber..

[57] Cunniff P.A. (1995). 942. 23, Thiamine (Vitamin B1) in Food, in AOAC Official Method of Analysis. AOAC Official Method of Analysis.

[58] (1973). Sodium Chloride for Industrial Use—Determination of the Loss of Mass at 110 Degrees C.

[59] Hrubša M., Siatka T., Nejmanová I., Vopršalová M., Krčmová L.K., Matoušová K., Javorská L., Macáková K., Mercolini L., Remião F. (2022). Biological Properties of Vitamins of the B-Complex, Part 1: Vitamins B1, B2, B3, and B5. Nutrients.

[60] Alibas I., Ipek S.S. (2026). Effects of Major Drying Methods on the Stability and Retention of Vitamin C, B Group Vitamins, Fat-Soluble Vitamins, and Carotenoids in Kiwifruits. J. Food Compos. Anal..

[61] Edwards K.A., Randall E.A., Wolfe P.C., Angert E.R., Kraft C.E. (2023). Dietary Factors Potentially Impacting Thiaminase I-Mediated Thiamine Deficiency. Sci. Rep..

[62] Schnellbächer A., Zimmer A. (2023). Stability and Requirement for Thiamin in a Cell Culture Feed Used to Produce New Biological Entities. Cells.

[63] Hiatt A.N., Ferruzzi M.G., Taylor L.S., Mauer L.J. (2008). Impact of Deliquescence on the Chemical Stability of Vitamins B1, B6, and C in Powder Blends. J. Agric. Food Chem..

[64] Edwards K.A., Tu-Maung N., Cheng K., Wang B., Baeumner A.J., Kraft C.E. (2017). Thiamine Assays—Advances, Challenges, and Caveats. ChemistryOpen.

[65] Tylicki A., Łotowski Z., Siemieniuk M., Ratkiewicz A. (2018). Thiamine and Selected Thiamine Antivitamins—Biological Activity and Methods of Synthesis. Biosci. Rep..

[66] Zaremba A., Waszkowiak K., Kmiecik D., Jędrusek-Golińska A., Jarzębski M., Szymandera-Buszka K. (2022). The Selection of the Optimal Impregnation Conditions of Vegetable Matrices with Iodine. Molecules.

[67] Ameye L., de Brouwer S., Gilliland D.L., Heckmann J., Janakiraman K., Kirwan B.A., Lavigne X., Moya F., Zammit V. (2025). Stability of Nutrients in Complex Liquid and Powder Food Matrices: Learnings from Shelf-Life Studies in Foods for Special Medical Purposes. Curr. Res. Food Sci..

[68] Voelker A.L., Miller J., Running C.A., Taylor L.S., Mauer L.J. (2018). Chemical Stability and Reaction Kinetics of Two Thiamine Salts (Thiamine Mononitrate and Thiamine Chloride Hydrochloride) in Solution. Food Res. Int..

[69] Ensom M.H.H., Decarie D. (2005). Stability of Thiamine in Extemporaneously Suspensions. Can. J. Hosp. Pharm..

[70] Roger D.D., Daoudou B., James B., Xavier E.F. (2014). Development of African Earthenware Container Imbedded with Nanosilver Particles for Food Preservation. J. Biomater. Nanobiotechnol..

[71] Piechocka J., Gramza-Michałowska A., Szymandera-Buszka K. (2021). The Changes in Antioxidant Activity of Selected Flavonoids and Caffeine Depending on the Dosage and Form of Thiamine. Molecules.

[72] McCoy R.E., Costa N.A., Morris A.E. (2015). Factors That Determine Stability of Highly Concentrated Chemically Defined Production Media. Biotechnol. Prog..

[73] Rodríguez García S.L., Raghavan V. (2022). Microwave-Assisted Extraction of Phenolic Compounds from Broccoli (*Brassica oleracea*) Stems, Leaves, and Florets: Optimization, Characterization, and Comparison with Maceration Extraction. Recent. Prog. Nutr..

[74] Liu M., Zhang L., Ser S.L., Cumming J.R., Ku K.M. (2018). Comparative Phytonutrient Analysis of Broccoli By-Products: The Potentials for Broccoli by-Product Utilization. Molecules.

[75] Gliszczynska-Swiglo A., Szymusiak H. (2006). Interaction of Food Flavonoids with Vitamins. Myricetin and Vitamin B1 as Model Compounds. Publ. Kyiv Natl. Univ. Trade Econ. Kiev. Ukr..

[76] Szymusiak H. (2002). Studies on the Effectiveness of Selected Antioxidants Found in Food Products.

[77] Musial C., Kuban-Jankowska A., Gorska-Ponikowska M. (2020). Beneficial Properties of Green Tea Catechins. Int. J. Mol. Sci..

[78] Cai Y.Z., Sun M., Xing J., Luo Q., Corke H. (2006). Structure-Radical Scavenging Activity Relationships of Phenolic Compounds from Traditional Chinese Medicinal Plants. Life Sci..

[79] He J., Xu L., Yang L., Wang X. (2018). Epigallocatechin Gallate Is the Most Effective Catechin against Antioxidant Stress via Hydrogen Peroxide and Radical Scavenging Activity. Med. Sci. Monit..

[80] Piechocka J., Szymandera-Buszka K. (2021). Thiamine in Lipid Systems vs. The Antioxidant Activity of Epigallocatechin Gallate and Caffeine. Sustainability.

[81] Kim M.G., Park J.H., Joo N. (2025). Analysis of Different Parts of Broccoli (*Brassica oleracea* Var. italica) Leaves and Retention of Functional Compounds Under Different Cooking Methods. Food Sci. Nutr..

[82] Szymandera-Buszka K. (2003). The Quantitative and Qualitative Changes of Thiamine in Sterilized Pork in the Presence of Selected Technological Additives. Pol. J. Food Nutr. Sci..

[83] El-Sawi S.A., Maamoun M.A.I., Farghaly A.A., Awad G.E.A. (2025). Exploring the Polyphenolic Content and Pharmacological Potential of Cauliflower Leaf Extract: A Combined Experimental and Computational Approach. South Afr. J. Bot..

[84] Martin C., Bellisle F. (1989). Eating Attitudes and Taste Responses in Young Ballerinas. Physiol. Behav..

[85] Zou T., Kang L., Yang C., Song H., Liu Y. (2019). Flavour Precursor Peptide from an Enzymatic Beef Hydrolysate Maillard Reaction-II: Mechanism of the Synthesis of Flavour Compounds from a Sulphur-Containing Peptide through a Maillard Reaction. LWT.

[86] Adrian J. (1974). Nutritional and Physiological Consequences of the Maillard Reaction. World Rev. Nutr. Diet..

[87] Mauron J. (1981). The Maillard Reaction in Food; a Critical Review from the Nutritional Standpoint. Prog. Food Nutr. Sci..

[88] Qi Y., Wang W., Yang T., Ding W., Xu B. (2025). Maillard Reaction in Flour Product Processing: Mechanism, Impact on Quality, and Mitigation Strategies of Harmful Products. Foods.

[89] Baranski R., Allender C., Klimek-Chodacka M. (2012). Towards Better Tasting and More Nutritious Carrots: Carotenoid and Sugar Content Variation in Carrot Genetic Resources. Food Res. Int..

[90] Vasile F.E., Simal S., Rosselló C., Eim V.S. (2022). Power Ultrasound-Assisted Impregnation of Apple Cubes with Vitamin B12. Food Bioprocess. Technol..

