# Supplementary material for: Analysis of the Use of Carrots, Cauliflower and Broccoli Waste Materials as a Matrix for Thiamine
Source: Foods. 2026 Feb 24;15(5):801. doi: 10.3390/foods15050801 (PMC12984307; doi:10.3390/foods15050801)
Supplement: Supplementary file 1 [file foods-15-00801-s001.zip › foods-4076351-supplementary.pdf]

Table S1. The thiamine content (%) during 230 days of storage of the dried thiamine hydrochloride fortified vegetables with various times of impregnation (20 and 60 minutes).

Temp	impregnations time [min]	Thiamine matrix	Days of storage					
			0	45	90	135	180	230
4⁰C	20	without	100.00	96.09	90.98	85.44	82.09	70.32
		crown of carrot	100.00	96.61	87.24	86.44	82.98	78.32
		peel of carrot	100.00	97.94	88.98	85.54	84.42	78.19
		peeled carrot	100.00	97.19	89.34	86.22	85.21	77.12
		stems of cauliflower	100.00	97.94	88.76	85.67	84.42	78.34
		florets of cauliflower	100.00	98.12	91.35	89.76	87.12	81.96
		leaves of cauliflower	100.00	98.53	92.45	91.23	90.56	82.67
		stems of broccoli	100.00	98.12	95.01	90.36	88.02	80.87
		florets of broccoli	100.00	98.89	97.03	92.37	89.32	82.12
		leaves of broccoli	100.00	98.99	96.02	93.11	90.01	85.02
	60	without	100.00	95.97	89.63	86.02	82.27	70.04
		crown of carrot	100.00	96.69	88.48	87.62	84.16	79.18
		peel of carrot	100.00	96.97	89.18	86.72	85.60	80.49
		peeled carrot	100.00	97.19	90.52	87.40	86.39	78.61
		stems of cauliflower	100.00	97.79	89.94	86.85	85.60	80.57
		florets of cauliflower	100.00	98.04	92.53	90.94	88.30	83.71
		leaves of cauliflower	100.00	98.66	93.63	92.41	91.74	84.95
		stems of broccoli	100.00	98.44	96.19	91.54	89.39	82.95
		florets of broccoli	100.00	98.49	98.21	93.55	90.50	83.58
		leaves of broccoli	100.00	98.94	97.20	94.29	91.19	86.57
21⁰C	20	without	100.00	95.69	90.08	84.14	75.87	67.42
		crown of carrot	100.00	95.83	86.25	84.88	81.00	76.17
		peel of carrot	100.00	97.16	87.99	83.98	82.44	76.04
		peeled carrot	100.00	96.41	88.35	84.66	83.23	74.97
		stems of cauliflower	100.00	97.16	87.77	84.11	82.94	76.90
		florets of cauliflower	100.00	97.34	90.36	88.20	85.14	79.81
		leaves of cauliflower	100.00	97.75	91.46	89.67	87.13	79.42
		stems of broccoli	100.00	97.34	94.02	88.80	86.04	78.72
		florets of broccoli	100.00	98.11	96.11	90.81	87.24	79.51
		leaves of broccoli	100.00	98.21	95.03	91.58	88.12	82.19
	60	without	100.00	95.05	90.42	84.18	76.03	67.08
		crown of carrot	100.00	96.09	86.59	85.62	81.66	76.43
		peel of carrot	100.00	97.42	88.33	84.72	83.10	76.30
		peeled carrot	100.00	96.67	88.70	85.40	83.89	75.23
		stems of cauliflower	100.00	97.42	88.11	84.85	83.60	77.16

		florets of cauliflower	100.00	97.60	90.67	88.94	85.80	80.07
		leaves of cauliflower	100.00	98.01	91.80	90.41	87.34	79.78
		stems of broccoli	100.00	97.60	94.36	89.54	86.70	78.98
		florets of broccoli	100.00	98.37	96.45	91.55	87.90	79.77
		leaves of broccoli	100.00	98.21	95.56	90.90	87.56	80.45
40^0C	20	without	100.00	88.54	81.65	71.36	60.19	42.34
		crown of carrot	100.00	94.84	83.51	76.02	70.14	59.23
		peel of carrot	100.00	96.17	85.25	75.12	71.58	60.06
		peeled carrot	100.00	95.42	85.64	75.80	72.31	59.76
		stems of cauliflower	100.00	96.17	85.03	75.25	72.08	59.45
		florets of cauliflower	100.00	96.35	87.62	79.34	74.28	63.83
		leaves of cauliflower	100.00	96.76	88.72	80.81	76.25	63.44
		stems of broccoli	100.00	96.35	91.24	79.91	75.18	62.74
		florets of broccoli	100.00	97.12	93.37	81.95	76.39	63.53
		leaves of broccoli	100.00	97.22	92.30	82.72	77.26	65.98
	60	without	100.00	88.02	81.3	71.34	58.95	41.24
		crown of carrot	100.00	95.15	84.05	77.01	70.90	60.12
		peel of carrot	100.00	96.48	85.79	76.11	72.34	60.95
		peeled carrot	100.00	95.73	86.18	76.79	73.07	60.71
		stems of cauliflower	100.00	96.48	85.60	76.24	72.84	60.34
		florets of cauliflower	100.00	96.66	88.16	80.33	75.04	64.72
		leaves of cauliflower	100.00	97.07	89.26	81.90	77.01	64.35
		stems of broccoli	100.00	96.66	91.80	80.90	75.94	63.63
		florets of broccoli	100.00	97.43	93.01	82.94	77.20	64.42
		leaves of broccoli	100.00	97.59	93.84	83.71	78.11	66.63

Table S2. The thiamine content (%) during 230 days of storage of the dried thiamine pyrophosphate forti-fied vegetables with various times of impregnation (20 and 60 minutes).

Temp	impregnations time [min]	Thiamine matrix	Days of storage					
			0	45	90	135	180	230
4^{0}C	20	without	100.00	94.06	88.95	83.41	80.06	68.29
		crown of carrot	100.00	94.58	85.21	84.41	80.95	76.29
		peel of carrot	100.00	95.91	86.92	83.51	82.39	76.34
		peeled carrot	100.00	95.16	87.31	84.19	83.18	75.09
		stems of cauliflower	100.00	95.91	86.73	83.64	82.39	76.31
		florets of cauliflower	100.00	96.65	89.31	87.73	85.09	79.93
		leaves of cauliflower	100.00	96.50	90.42	89.20	88.53	80.64
		stems of broccoli	100.00	96.09	92.98	88.33	85.76	78.84
		florets of broccoli	100.00	96.84	95.00	90.34	87.29	80.09
		leaves of broccoli	100.00	95.98	93.99	91.18	87.98	83.12
	60	without	100.00	94.15	88.42	83.09	67.99	70.65
		crown of carrot	100.00	94.67	85.68	85.09	81.74	76.97
		peel of carrot	100.00	96.00	87.39	84.19	82.45	77.01
		peeled carrot	100.00	95.25	87.78	84.93	83.86	75.77
		stems of cauliflower	100.00	96.00	87.20	84.32	83.07	75.74
		florets of cauliflower	100.00	96.74	89.78	88.41	85.89	80.99
		leaves of cauliflower	100.00	96.59	90.89	89.86	89.21	81.32
		stems of broccoli	100.00	96.18	93.45	89.01	86.44	79.52
		florets of broccoli	100.00	96.93	95.47	91.02	87.97	82.93
		leaves of broccoli	100.00	97.35	94.46	91.86	88.66	83.80
21^{0}C	20	without	100.00	93.31	86.2	80.86	70.01	58.54
		crown of carrot	100.00	93.84	84.26	82.89	79.01	74.17
		peel of carrot	100.00	95.17	86.00	81.99	80.45	74.05
		peeled carrot	100.00	94.45	86.35	82.67	81.24	72.98
		stems of cauliflower	100.00	95.12	85.78	82.12	80.95	74.91
		florets of cauliflower	100.00	95.34	88.35	86.21	83.16	77.81
		leaves of cauliflower	100.00	95.80	89.46	87.68	85.14	77.43
		stems of broccoli	100.00	95.32	92.03	86.81	84.05	76.73
		florets of broccoli	100.00	96.13	94.12	88.82	85.26	77.52
		leaves of broccoli	100.00	96.21	93.06	89.55	86.13	80.21
	60	without	100.00	93.05	86.18	79.72	70.08	58.55
		crown of carrot	100.00	94.08	84.64	83.75	79.70	75.18
		peel of carrot	100.00	95.03	86.38	82.85	81.22	75.06
		peeled carrot	100.00	94.70	86.73	83.53	82.01	73.87
		stems of cauliflower	100.00	95.36	86.16	82.98	81.70	75.92

		florets of cauliflower	100.00	95.58	88.73	87.07	83.93	78.82
		leaves of cauliflower	100.00	96.03	89.84	88.50	85.91	78.44
		stems of broccoli	100.00	95.56	92.41	87.67	84.82	77.74
		florets of broccoli	100.00	96.37	93.98	89.70	86.05	78.53
		leaves of broccoli	100.00	96.43	94.12	90.41	86.90	81.04
40^{0}C	20	without	100.00	82.11	67.38	49.35	43.32	25.31
		crown of carrot	100.00	92.85	81.52	74.03	68.15	57.24
		peel of carrot	100.00	94.18	81.30	71.16	67.62	55.04
		peeled carrot	100.00	93.43	81.66	71.84	68.35	55.60
		stems of cauliflower	100.00	94.18	81.05	71.29	68.60	53.10
		florets of cauliflower	100.00	94.36	83.64	75.38	70.12	59.54
		leaves of cauliflower	100.00	94.68	84.74	76.85	72.29	59.15
		stems of broccoli	100.00	94.36	87.03	75.95	71.22	58.45
		florets of broccoli	100.00	96.75	89.83	77.99	72.43	59.60
		leaves of broccoli	100.00	96.12	88.32	78.76	73.30	61.69
	60	without	100.00	82.19	66.87	48.43	42.8	25.39
		crown of carrot	100.00	93.93	82.60	75.11	69.06	58.32
		peel of carrot	100.00	95.26	82.38	72.20	68.70	56.12
		peeled carrot	100.00	94.51	82.74	72.94	69.43	56.68
		stems of cauliflower	100.00	95.26	82.26	72.37	69.45	54.18
		florets of cauliflower	100.00	95.44	84.72	76.46	71.20	60.65
		leaves of cauliflower	100.00	95.75	85.82	77.93	73.37	60.27
		stems of broccoli	100.00	95.44	88.18	77.03	72.30	59.53
		florets of broccoli	100.00	96.01	89.18	79.32	73.51	61.54
		leaves of broccoli	100.00	96.32	89.40	79.84	74.39	62.77
